# Gut–prostate axis in prostate cancer: microbiome signatures, mechanistic insights, and therapeutic opportunities

**DOI:** 10.3389/fcimb.2026.1882172

**Published:** 2026-09-08

**Authors:** Kenan Wang, Qizhen Tang, Weiwei Fan, Quanxin Su

**Affiliations:** Department of Urology, The First Affiliated Hospital of Dalian Medical University, Dalian, China

**Keywords:** androgen receptor, gut microbiota, gut-prostate axis, microbial metabolites, prostate cancer

## Abstract

Prostate cancer (PCa) is one of the most common malignant tumors in men, and its onset and progression may be closely associated with an imbalance in the gut microbiota. Existing studies indicate that PCa patients exhibit reduced fecal microbiota diversity and altered microbiota composition, with the abundance of certain bacterial genera correlated with disease risk and progression. Mechanistically, the gut microbiota may regulate signaling pathways such as IGF-1, MAPK/PI3K, and NF-κB through metabolites including short-chain fatty acids, bile acids, and microbiota-derived androgens, thereby potentially contributing to tumor proliferation, inflammatory responses, and immune evasion. Androgen deprivation therapy (ADT) can also reshape the gut microbiota; certain bacterial populations may contribute to the development of castration resistance through androgen metabolism, while changes in bacteria such as *Akkermansia muciniphila* are associated with treatment response. Currently, strategies such as dietary interventions, probiotics, and fecal microbiota transplantation have shown some promise; however, most existing studies are small-sample or cross-sectional in nature, and causal relationships remain unclear. Future studies should combine multicenter longitudinal cohorts with multi-omics technologies to further elucidate the translational value of the gut microbiota in PCa screening, risk stratification, prognostic assessment, treatment-response prediction, toxicity monitoring, and personalized microbiome-targeted interventions.

## 1 Introduction

Prostate cancer (PCa) is one of the most common malignancies in men, affecting hundreds of thousands of men worldwide each year and ranking among the leading causes of cancer-related mortality in men (Bray et al., 2024). Although radical surgery and radiation therapy achieve high cure rates in early-stage disease, a significant proportion of patients still experience recurrence within ten years (Shore et al., 2024). In metastatic disease, the androgen receptor (AR) signaling pathway is a major driver; consequently, androgen deprivation therapy (ADT) has become the standard treatment. However, most patients eventually progress to castration-resistant prostate cancer (CRPC) (Jacob et al., 2021). The development of PCa is influenced by various factors, including age, genetics, diet, obesity, and a history of inflammation (Bergengren et al., 2023). Notably, modifiable factors such as diet, obesity, and chronic inflammation are all closely associated with the gut microbiota. Consequently, the concept of the “gut-prostate axis” has garnered increasing attention, suggesting that the gut microbiota may influence prostate tumor initiation, progression, and treatment response by producing metabolites and modulating the host’s endocrine and immune environments (Fujita et al., 2023). Based on this theoretical framework, investigating whether gut microbiota dysbiosis serves as a common cause, a risk marker, or a potential therapeutic target for the development and progression of PCa has become a major research focus in this field in recent years (Huang et al., 2024; Laaraj et al., 2025).

The gut microbiota is a vital component of the host’s endogenous environment; its metabolites and signaling molecules can enter the systemic circulation and influence the immune, metabolic, and endocrine functions of distant organs (Wikoff et al., 2009; Rooks and Garrett, 2016; Schroeder and Backhed, 2016). Existing research indicates that the gut microbiota participates in regulating systemic immune, metabolic, and endocrine balance, exerting a significant impact on host health. When the gut microbiota is dysbiotic, levels of pro-inflammatory or pro-tumorigenic metabolites such as lipopolysaccharide (LPS) and bile acids increase, while the production and function of beneficial metabolites such as short-chain fatty acids (SCFAs) may be disrupted, thereby potentially contributing to chronic inflammation, immune dysregulation, and the formation of a tumor-associated microenvironment (Yang and Cong, 2021; Li et al., 2022). Recent research on the tumor microbiome has further highlighted that bacteria may participate in tumor initiation, promotion, and progression. In common solid tumors such as colorectal cancer and liver cancer, the gut microbiota has been shown to participate in carcinogenesis and modulate responses to anticancer therapies, including chemotherapy, targeted therapy, and immunotherapy (de Martel et al., 2012; Ting et al., 2022; El Tekle and Garrett, 2023). Regarding PCa, preliminary reports suggest that the gut microbiota may influence androgen metabolism, chronic inflammation levels, and the tumor immune microenvironment, thereby potentially contributing to tumor initiation and progression (Golombos et al., 2018). Although promising associations have been established between the gut microbiota and PCa, this field remains in its infancy. Against this backdrop, this review focuses on the “gut-prostate axis,” evaluating the current evidence and covering topics such as the microbiome characteristics of PCa patients, the core mechanisms underlying microbiome effects, bidirectional interactions between the microbiome and treatment, and microbiome intervention strategies, with the aim of clarifying how microbiome-related findings may be translated into PCa risk stratification, biomarker development and treatment-response prediction.

## 2 Gut microbiome characteristics in PCa patients

In recent years, the relationship between the gut microbiota and prostate cancer (PCa) has gradually garnered attention. Existing clinical studies primarily employ 16S rRNA sequencing using fecal or rectal swab samples, with a few studies utilizing shotgun metagenomic sequencing. From a methodological perspective, 16S rRNA sequencing is cost-effective and suitable for exploratory cohort studies, but it has limited taxonomic resolution and usually relies on predicted rather than directly measured microbial functions. In contrast, shotgun metagenomic sequencing can provide higher species- or strain-level resolution and functional pathway information, but it is more expensive and more sensitive to sequencing depth, reference databases, and bioinformatic pipelines. Overall, there are certain differences in gut microbiota composition between PCa patients and healthy individuals, patients with benign prostatic diseases, or those with negative biopsy results. However, due to inconsistencies across studies regarding sample types, control selection, sequencing platforms and patient clinical stratification, a highly unified “PCa-specific microbiome profile” has not yet been established. For direct comparison across these heterogeneous clinical studies, **Supplementary Table 1** summarizes the study population and grouping, sample type and analytical method, α- and β-diversity findings, and principal differential taxa.

Early studies suggest that certain anaerobic bacteria and inflammation-associated microbiota in the gut microbiota of PCa patients may undergo changes. Golombos et al. used shotgun metagenomic sequencing to compare patients with intermediate- to high-risk localized PCa with controls with benign prostatic disease and found that the relative abundance of *Bacteroides massiliensis* was elevated in PCa patients, while the abundance of *Faecalibacterium prausnitzii* and *Eubacterium rectale* was higher in the control group, suggesting that a reduction in SCFA-producing bacteria may be associated with PCa (Golombos et al., 2018). Liss et al. analyzed 16S rRNA sequencing data from rectal swabs in a prostate biopsy cohort and found that while α-diversity was generally similar between PCa patients and biopsy-negative controls, β-diversity showed significant differences; the PCa group exhibited enrichment of *Bacteroides* and *Streptococcus*, and functional prediction suggested potential alterations in folate and arginine metabolic pathways (Liss et al., 2018). Smith et al., however, found that both the Chao1 and Observed Species indices were higher in 8 PCa patients than in the control group (Smith et al., 2021). These results suggest that PCa-associated microbiota changes may be more reflected in alterations in community structure and the abundance of specific genera, rather than necessarily manifesting as a significant overall decrease in α-diversity.

However, not all studies have observed consistent differences in the gut microbiota. Alanee et al. analyzed both fecal and urinary microbiota in PCa patients and non-cancerous/benign controls and found that differences in the Shannon index of fecal samples were not significant, and the fecal microbiota could not be clearly separated between PCa and non-PCa groups (Alanee et al., 2019). Katz et al. compared newly diagnosed PCa patients with patients with benign prostatic hyperplasia and also found no significant differences in gut microbiota diversity between the two groups, nor were there consistent differences across different Gleason or ISUP stratifications (Katz et al., 2022). Tsai et al. included PCa, benign prostatic hyperplasia, and control groups; their results similarly showed no significant differences in fecal samples among the three groups, while differences in urinary microbiota were relatively more pronounced (Tsai et al., 2022). These negative or inconsistent results suggest that the association between PCa and the gut microbiota may be highly context-dependent and influenced by multiple sources of heterogeneity, including sample size, control group definition, disease stage, tumor aggressiveness, diet, BMI, medication history, antibiotic exposure, prior or ongoing cancer treatment, and regional differences. In addition, studies using fecal samples, rectal swabs, urinary samples, or prostate tissue may capture different microbial niches, which may partly explain why some studies identify clear fecal microbial differences whereas others observe more pronounced urinary or tissue-associated changes.

From the perspective of high-risk PCa or tumor progression, some studies suggest that specific microbial communities may be associated with tumor aggressiveness. Matsushita et al. compared high-risk PCa with biopsy-negative/low-risk PCa groups among 152 patients with suspected PCa, finding elevated abundances of Rikenellaceae, *Alistipes*, and *Lachnospira* in high-risk PCa patient (Matsushita et al., 2021b). They constructed a microbiome index based on 18 bacterial genera, which demonstrated potential superior to prostate-specific antigen (PSA) in identifying high-risk PCa (Matsushita et al., 2021b). From a translational perspective, such microbiome-based indices may be most clinically useful when integrated with PSA, multiparametric magnetic resonance imaging (mpMRI) findings, Gleason/ISUP grade, biopsy status, and blood-based biomarkers, rather than being used as standalone diagnostic tools. Zhong et al. further compared patients with benign prostatic hyperplasia, non-metastatic PCa, and metastatic PCa, finding elevated Proteobacteria abundance in patients with metastatic PCa, which was associated with IL-6 levels, lymph node metastasis, and distant metastasis (Zhong et al., 2022). These studies suggest that changes in the gut microbiota may not only be associated with the development of PCa but may also be involved in PCa progression, inflammatory responses, and metastatic processes.

More recent studies further support the presence of specific microbial composition and functional characteristics in PCa patients. Kalinen et al. found in a population of patients undergoing biopsy for suspected PCa that there were no significant differences in Chao-1 and Shannon indices between PCa patients and benign cases (Kalinen et al., 2024). However, Bray-Curtis distance analysis revealed significant differences in β-diversity;changes in bacterial communities such as *Prevotella* 9 were observed in PCa patients, and functional predictions suggested potential differences in pathways including 5α-reductase, copper absorption, and retinol metabolism. Cocomazzi et al. compared newly diagnosed, untreated PCa patients with healthy controls and found that the PCa group had significantly reduced α-diversity and richness, as well as significantly different β-diversity (Cocomazzi et al., 2025). In PCa patients, *Lactobacillus*, *Prevotella* 9, *Anaerostipes*, *Alistipes finegoldii*, and *Bacteroides fragilis* were elevated, while *Collinsella*, *Coprococcus*, *Butyricimonas*, and *Fusicatenibacter* were reduced, suggesting that PCa patients may exhibit a characteristic reduction in SCFA-producing bacteria and an increase in potentially pro-inflammatory microbiota (Cocomazzi et al., 2025). Furthermore, differences in the gut microbiota of PCa patients across various clinical contexts suggest that microbial profiles may be associated with disease stage, treatment status, and risk of progression (Carballo Quinta et al., 2025). In recent years, several Mendelian randomization studies have also suggested, from the perspective of genetic causality inference, a potential association between the gut microbiota and PCa risk (Xie and Hu, 2023; Wang et al., 2024), and further proposed that immune cells, sex hormones, blood metabolites, and proteins may play mediating roles in the “gut-prostate” axis (Li et al., 2024; Yue et al., 2024; Liu et al., 2025).

Taken together, the available microbiome composition studies should be interpreted with caution. The inconsistent findings across studies are likely attributable not only to biological heterogeneity but also to substantial methodological differences, including sample source, control group definition, sequencing strategy, dietary habits, medication exposure, disease stage, and treatment status. Therefore, a universal PCa-specific gut microbiome signature has not yet been established. Nevertheless, several recurring patterns can be identified across independent studies, including enrichment of *Bacteroides*, *Streptococcus*, *Alistipes*/Rikenellaceae, *Prevotella*-related taxa, and Proteobacteria in some PCa or aggressive PCa cohorts, together with reduced abundance of SCFA-producing bacteria such as *Faecalibacterium prausnitzii* and *Eubacterium rectale* in others. These taxa should currently be regarded as candidate microbial markers rather than validated diagnostic or prognostic biomarkers. Future studies should use standardized sampling and sequencing protocols, well-defined control groups, and independent validation cohorts to distinguish robust disease-associated taxa from study-specific signals.

## 3 Mechanisms by which the gut microbiome may influence the pathogenesis of PCa

### 3.1 Direct/indirect effects of microbial metabolites

The influence of the gut microbiota on host reproductive function may be mediated through its metabolites and its ability to reshape the host’s metabolic network. Among these, short-chain fatty acids (SCFAs, primarily acetic, propionic, and butyric acids) are metabolites produced by gut bacteria through the fermentation of dietary fiber and are known to exert regulatory effects in various cancers (Koh et al., 2016; Li et al., 2023; Hou et al., 2024). However, their role in PCa may be context-dependent. In a study by Matsushita et al., antibiotic intervention in Pten-knockout PCa mice reduced the levels of Rikenellaceae, Clostridiales, and other bacterial groups, as well as fecal SCFA levels; it also decreased circulating and local prostate IGF1 expression and inhibited MAPK/PI3K signaling; whereas SCFA supplementation restored IGF1 levels and was associated with increased tumor growth in this model, suggesting that the “gut microbiota-SCFA-IGF1-MAPK/PI3K” axis may contribute to PCa cell proliferation (Matsushita et al., 2021a). On the other hand, Liu et al. found that FMT from CRPC patients increased levels of SCFA-producing bacteria such as *Ruminococcus*, *Alistipes*, and *Phascolarctobacterium*, as well as levels of acetic acid and butyric acid (Liu et al., 2023). SCFAs further enhance PCa cell migration, invasion, and tumor microenvironment remodeling by inducing Toll-like receptor 3 (TLR3)-mediated autophagy, activating NF-κB/MAPK signaling, and promoting CCL20 release and M2 macrophage polarization.

Several factors may help explain these apparently discordant findings. First, SCFA effects may be concentration- and exposure-dependent. Physiological concentrations generated continuously through dietary-fiber fermentation may not be equivalent to the relatively high doses used in supplementation or cell-culture experiments. Second, acetate, propionate, and butyrate should not necessarily be considered functionally interchangeable, because they differ in absorption, tissue distribution, receptor engagement, and intracellular metabolism. Third, disease stage and treatment context may alter the biological response. Tumors with preserved androgen sensitivity may respond differently from tumors exposed to long-term ADT or established CRPC, in which AR reactivation, metabolic adaptation, and immune remodeling are more prominent. Finally, differences in animal models, supplementation dose, administration route, treatment duration, and baseline microbiota may also contribute to the observed inconsistency. Therefore, the role of SCFAs in PCa cannot be simply regarded as uniformly protective or tumor-promoting; their specific effects may differ according to tumor stage, host metabolic status, dietary substrate availability, baseline microbial community structure, androgen-deprivation status, and the tumor immune microenvironment.

Fatty acid and vitamin-related pathways may also be involved in the “gut-prostate axis.” Lachance et al. found that FMT from patients with high tumor burden PCa was associated with increased PCa growth in mice, whereas omega-3 MAG-EPA intervention reduced PCa growth and progression, accompanied by changes in Ruminococcaceae and fecal butyrate levels, suggesting that long-chain fatty acid metabolism and microbiota-derived butyrate may jointly participate in regulating PCa progression (Lachance et al., 2024). In the study by Hu et al., Icaritin restored levels of, *Akkermansia*ceae, and vitamin K2, improved the profile of adipokines such as leptin and adiponectin, and prolonged survival in TRAMP mice, suggesting that the “*Akkermansia*ceae-vitamin K2-adipokine” axis may be involved in inhibiting high-fat diet-associated PCa progression (Hu et al., 2026).

Furthermore, studies have found that dietary factors may influence PCa progression by reshaping the gut microbiota and its metabolites. Liu et al. found that exposure to a high-fat diet can alter the levels of bacterial groups such as *Lachnospiraceae*, *Roseburia*, and *Amycolatopsis*, as well as serum metabolite levels, thereby affecting PCa incidence and tumor burden in TRAMP mice (Liu et al., 2019). Further studies revealed that a lard-rich diet can induce changes in gut microbiota, including Clostridiales and Lactobacillales, accompanied by the reprogramming of genes related to lipid metabolism and cholesterol biosynthesis in PCa tissue, suggesting that the “dietary fat-gut microbiota-lipid metabolism” axis may be involved in PCa progression (Sato et al., 2022). In high-fat diet-associated PCa models, gut microbiota dysbiosis has been reported to increase serum LPS levels, which may contribute to tumor growth through TLR4, mast cell/histamine H1 receptor, and IL-6/STAT3 inflammatory signaling pathways (Matsushita et al., 2022). These findings suggest that Westernized or high-fat diets may provide a tumor-promoting environment for PCa progression by inducing gut microbiota imbalance, metabolic abnormalities, and systemic inflammation. Importantly, these metabolic effects may converge with inflammatory and immune pathways: altered SCFA, lipid, vitamin K2, and cholesterol metabolism can influence IGF-1, MAPK/PI3K, NF-κB, IL-6/STAT3, and AR-related signaling, thereby linking dietary dysbiosis to endocrine regulation, macrophage polarization, and tumor immune remodeling.

### 3.2 Intestinal androgen synthesis promotes castration resistance

Gut microbiota-mediated alterations in androgen metabolism may represent a potential mechanism associated with PCa progression, particularly CRPC. Certain gut microbial populations may synthesize or convert hormonal precursors, thereby potentially influencing systemic levels of active androgens and subsequently affecting prostate tumors. In the study by Pernigoni et al., ADT promoted the expansion of gut microbiota associated with CRPC in both mice and humans, some of which possess the ability to convert androgen precursors into active androgens; FMT derived from CRPC patients rendered mouse tumors resistant to castration, whereas FMT derived from human spermatogonial stem cells (HSPCs) or intervention with *Prevotella stercorea* suppressed tumor growth (Pernigoni et al., 2021). Daisley et al. also found that ADT reduces androgen-utilizing *Corynebacterium* spp., while abiraterone treatment enriches *Akkermansia muciniphila* and enhances functions related to vitamin K2 biosynthesis (Daisley et al., 2020). Furthermore, ADT treatment itself reduces α-diversity and alters β-diversity in the fecal microbiota of PCa patients (Li et al., 2021). Longitudinal studies have also demonstrated dynamic changes in the gut microbiota and metabolome following ADT (Kure et al., 2023), and long-term ADT is associated with an increase in bacterial genera involved in testosterone synthesis (Wang, 2024). Comparisons between HSPC and CRPC patients suggest that differences in microbial communities, such as those involving Firmicutes, *Gemella* and *Lactobacillus*, exist at the CRPC stage. This finding has been partially validated in mouse models (Fujimoto et al., 2025). Recent studies on bacterial androgen-producing pathways have further revealed that the desF gene in the gut bacterium *Clostridium scindens* may be involved in androgen metabolism. Elevated desF levels in fecal samples were observed in PCa patients who did not respond to abiraterone/prednisone therapy, and bacteria originating from the urinary tract or prostate may also promote AR-dependent PCa cell proliferation through steroid metabolism (Wang et al., 2025). These findings suggest that the gut microbiota may serve as an alternative source of androgens, promoting endocrine resistance, and that different androgen axis treatment modalities can reshape the gut microbiota and metabolic function. Bacteria in the gut and urogenital tract may jointly participate in androgen production and drug metabolism, thereby influencing the response to endocrine therapy. Furthermore, in a study of high-risk localized PCa patients, post-ADT changes in the gut microbiota were associated with dietary habits and total testosterone levels, further suggesting that treatment, diet, and endocrine status may collectively shape the gut microbiome (Ishida et al., 2026). This endocrine mechanism may also interact with inflammatory and immune pathways, because ADT-induced microbial remodeling can alter metabolite production, intestinal barrier integrity, LPS exposure, and cytokine signaling, which may in turn influence AR activity, tumor-associated inflammation, and immune surveillance during the transition toward CRPC. Among the proposed mechanisms, microbiota-mediated androgen biosynthesis has relatively coherent perturbation-based support from bacterial-transfer and intervention experiments in castrated mouse models. In humans, however, the available findings mainly demonstrate associations with ADT exposure, CRPC status, androgen-related microbial functions, and treatment response, rather than direct causality.

### 3.3 Inflammatory responses and intestinal barrier disruption

Inflammatory responses and intestinal barrier disruption are also potential pathways through which the gut microbiota may contribute to PCa progression. Zhong et al. found that antibiotic-induced gut dysbiosis was associated with Proteobacteria enrichment, increased intestinal permeability, and elevated LPS, which may activate the NF-κB-IL6-STAT3 signaling pathway and thereby contribute to PCa proliferation, progression, and docetaxel resistance; the abundance of Proteobacteria in clinical samples is also associated with IL-6 levels, lymph node metastasis, and distant metastasis (Zhong et al., 2022). Lin et al. further demonstrated in a CRPC animal model that CRPC-associated dysbiosis may promote PCa cell proliferation, migration, and invasion while inhibiting apoptosis by activating the TNF-α-mediated TLR4/MyD88/NF-κB pathway; FMT from healthy mice, however, slowed CRPC tumor growth and reduced TNF-α levels (Lin et al., 2024). Furthermore, treatment with Astragaloside IV-PESV in PCa mice restored gut microbiota and metabolic homeostasis and downregulated the expression of RAGE, NF-κB, TNF-α, and IL-6, suggesting that the AGE/RAGE inflammatory pathway may also be a key node through which the microbiota regulates PCa progression (You et al., 2024). These inflammatory pathways are mechanistically linked to both endocrine resistance and immune escape: NF-κB and IL-6/STAT3 signaling can interact with AR signaling, promote macrophage polarization, and regulate PD-L1-related immune suppression, thereby connecting barrier dysfunction with tumor progression and treatment resistance. The barrier dysfunction/LPS–TLR4/MyD88/NF-κB–IL-6/STAT3 pathway has been functionally supported by antibiotic, FMT, and pathway-intervention experiments in animal models. By contrast, the human evidence linking Proteobacteria abundance with IL-6, metastasis, and treatment resistance remains primarily observational.

### 3.4 Regulation of antitumor immune surveillance

The gut microbiota can also influence the response to PCa treatment by modulating antitumor immunity. Terrisse et al. found in PCa mice and 65 patients with HSPC/CRPC that *A. muciniphila* is reduced in the gut under PCa conditions, while ADT can reverse this change; antibiotic-induced depletion of the gut microbiota weakens ADT efficacy, whereas co-culturing or oral administration of *Akkermansia* can enhance the antitumor effects of ADT, suggesting that the gut microbiota may regulate ADT efficacy through thymic output and T-cell immunity (Terrisse et al., 2022). Yu et al. further found that *A. muciniphila* is enriched in ADT-sensitive CRPC patients; its metabolite inosine can improve the intestinal barrier, reduce LPS levels, and inhibit castration resistance via the LPS/NF-κB/AR axis (Yu et al., 2024). In studies on the mechanisms of traditional Chinese medicine formulations, ICA-CUR was found to inhibit PCa progression by modulating the gut microbiota and SCFAs, influencing the DNMT1/IGFBP2/EGFR/STAT3/PD-L1 axis, and enhancing the antitumor immunity of CD8+ T cells (Xu et al., 2024).Additionally, the gut-derived metabolites 16(R)-HETE and 6-keto-PGE1 have been found to be associated with PD-L1 on exosomes and can enhance the efficacy of anti-PD-L1 therapy in animal models (Liu et al., 2026). These findings suggest that immune regulation is closely integrated with metabolic, inflammatory, and endocrine mechanisms. For example, *A. muciniphila* and inosine may improve intestinal barrier function and reduce LPS/NF-κB signaling, thereby indirectly affecting AR activation and castration resistance, while SCFA-related signaling may influence macrophage polarization, PD-L1 expression, and CD8+ T-cell activity.

### 3.5 Non-bacterial components: fungi and viruses

Although most PCa microbiome studies have focused on bacteria, fungi and viruses may also contribute to cross-kingdom microbial interactions. PCa-specific evidence, however, remains limited. Differences in the circulating fungal profile have been reported between patients with PCa and healthy controls (Wang et al., 2022), and exploratory sequencing of prostate tissue has detected fungal signals and ochratoxin A-related findings (Fry et al., 2022). These studies were small and affected by limitations including the absence of appropriate tissue controls, low microbial biomass, and uncertainty in taxonomic confirmation; therefore, they do not establish a causal role for fungi in PCa. Fungal cell-wall components or metabolites could theoretically influence intestinal-barrier integrity and systemic immune signaling, but these mechanisms have mainly been demonstrated in other tumor types. Viral evidence should also be interpreted according to viral origin. An increased immune response to a human endogenous retrovirus K GAG protein has been associated with advanced PCa (Reis et al., 2013), but endogenous retroviral expression is distinct from the gut virome. Direct evidence linking gut bacteriophage composition to PCa initiation, progression, or treatment resistance is currently insufficient. Bacteriophages may nevertheless indirectly influence the gut–prostate axis by reshaping bacterial communities, transferring functional genes, or modifying microbial metabolite production. Future studies should integrate bacterial, fungal, and viral profiling and incorporate stringent contamination controls, absolute quantification, longitudinal sampling, and functional validation before assigning disease relevance to low-abundance non-bacterial signals.

**Figure 1:**
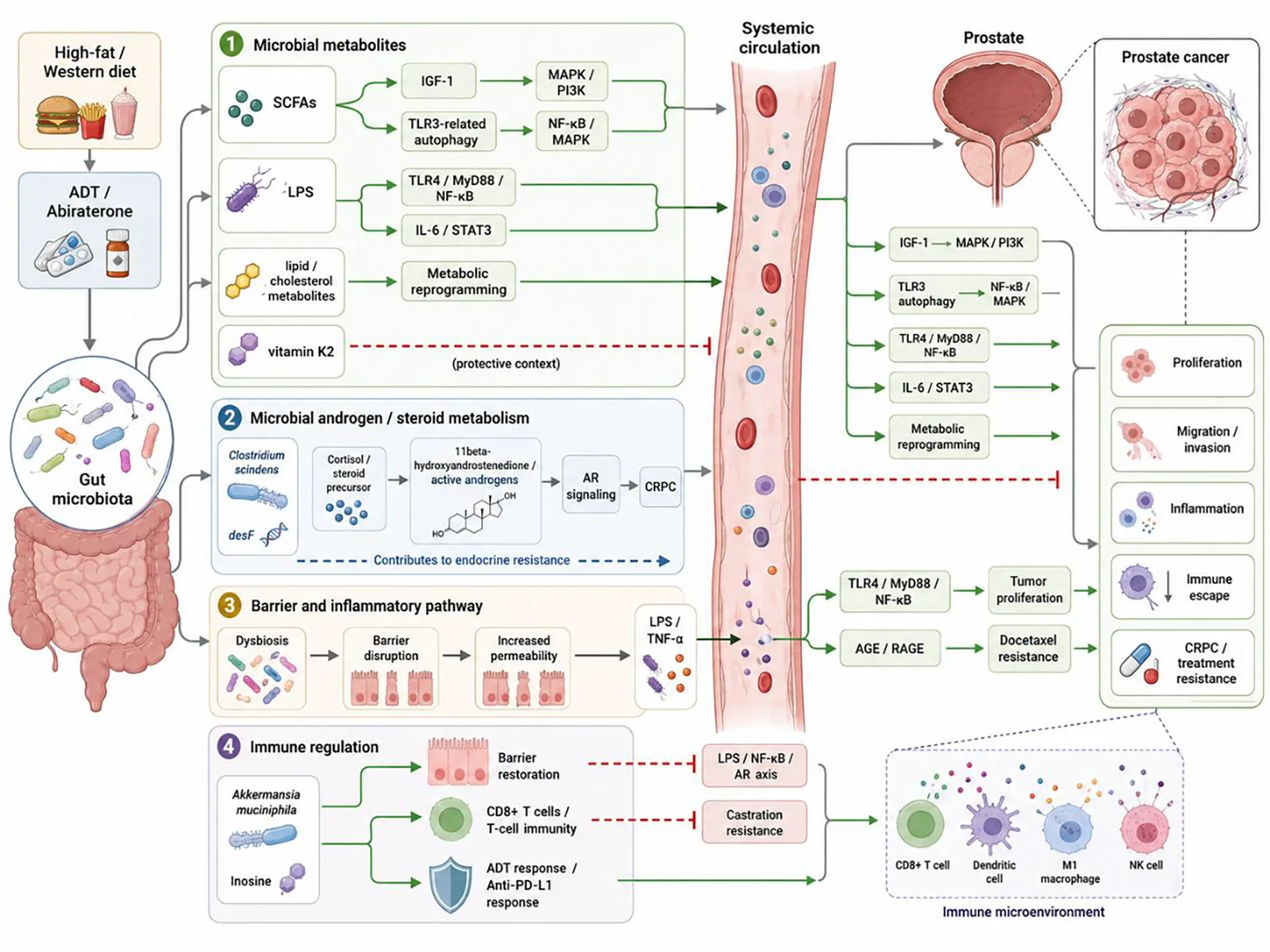
Integrated mechanisms linking the gut microbiota to prostate cancer. Metabolic, inflammatory, barrier-related, and immune pathways may operate across both hormone-sensitive and castration-resistant disease, whereas microbiota-mediated androgen synthesis and AR reactivation are particularly relevant during ADT and progression toward CRPC. Prostate cancer treatment may reshape the gut microbiota, while microbiota-derived metabolites may in turn influence treatment response, representing a bidirectional interaction.

Overall, the gut microbiota may influence PCa onset and progression through an interconnected metabolic–endocrine–inflammatory–immune network (**Figure 1**). These mechanisms should not be interpreted as being uniformly stage-specific: metabolic, inflammatory, barrier-related, and immune pathways may operate across different stages of PCa, whereas microbiota-mediated androgen synthesis and AR reactivation appear to be particularly relevant during ADT and progression toward CRPC. In this framework, microbial metabolites such as SCFAs, fatty acids, vitamin K2, cholesterol-related metabolites, and microbiota-derived androgens may reshape host metabolism and endocrine signaling, thereby affecting IGF-1, MAPK/PI3K, AR, and STAT3-related pathways. At the same time, intestinal barrier disruption and increased LPS exposure may activate TLR4/MyD88/NF-κB and IL-6/STAT3 signaling, which can further interact with AR signaling, macrophage polarization, PD-L1 expression, and CD8+ T-cell-mediated antitumor immunity. However, the strength of evidence differs substantially among these mechanisms. The androgen biosynthesis and inflammatory pathways are supported by relatively coherent experimental evidence, including FMT, antibiotic intervention, and mechanistic animal models, whereas the roles of SCFAs and other microbial metabolites appear more context-dependent. For example, SCFAs may exert tumor-promoting or tumor-suppressive effects depending on tumor stage, host metabolic status, microbial community structure, and the immune microenvironment. Methodologically, these studies are affected by differences in animal strain, tumor model, antibiotic regimen, FMT donor selection, colonization efficiency, metabolite dose, treatment duration, and endpoint selection, which may limit direct extrapolation to human PCa. Therefore, these mechanisms should not be interpreted as linear or universally applicable pathways. Most current evidence remains preclinical or translational, and causal validation in humans is still limited. Future studies should prioritize strain-level identification, metabolite quantification, temporal sampling, and intervention-based validation to determine whether these mechanisms are drivers of PCa progression or secondary consequences of tumor- and treatment-associated dysbiosis.

From a stage-oriented perspective, PCa initiation or early progression and treatment-resistance progression share a background of dysbiosis-associated metabolic alteration, intestinal barrier dysfunction, inflammation, and immune remodeling, but differ in their relative mechanistic emphasis. In localized or untreated PCa, recurring candidate patterns include enrichment of *Bacteroides*, *Streptococcus*, *Alistipes*/Rikenellaceae, *Prevotella*-related taxa, or Proteobacteria in some cohorts, together with depletion of certain SCFA-producing bacteria; the proposed mechanisms mainly involve diet–microbiota metabolism, SCFA–IGF-1–MAPK/PI3K signaling, and LPS-related chronic inflammation. During ADT exposure and progression toward CRPC, the more distinctive feature appears to be treatment-selected microbial function rather than a reproducible single taxon, particularly steroid conversion and androgen biosynthesis that may reactivate AR signaling, together with inflammatory and immune pathways affecting treatment response. For example, *C. scindens* has been linked to microbial androgen production, whereas *A. muciniphila* and *P. stercorea* have been associated in some contexts with treatment sensitivity or tumor suppression. Thus, taxonomic abundance cannot be assigned a uniform functional meaning across disease stages. Initiation-related human evidence remains mainly associative, whereas resistance-related mechanisms have stronger perturbation-based support in animal models but still lack direct causal validation in patients.

## 4 Bidirectional interactions between the gut microbiome and prostate cancer treatment

There is a dynamic interaction between PCa treatment (especially systemic therapy) and the gut microbiota. On the one hand, tumor treatment can significantly alter the microbial community structure; on the other hand, the composition of the microbiota may influence treatment efficacy. As mentioned earlier, certain bacterial strains activate androgen biosynthesis pathways following ADT; abiraterone therapy also alters the microbiota, enriching *A. muciniphila*. Radiotherapy has a more direct impact on the intestinal mucosa. Danckaert et al. conducted longitudinal fecal microbiome and metabolomic analyses in 50 patients undergoing radiotherapy to the prostate or prostate bed, finding that gut microbiome β-diversity changed significantly during radiotherapy, suggesting that radiotherapy can induce disturbances in the gut microbiome (Danckaert et al., 2023). Jang et al. found that among PCa patients undergoing radical radiotherapy, those who developed acute gastrointestinal toxicity exhibited lower α-diversity and an elevated microbial community polarization index during radiotherapy, suggesting that radiotherapy-induced dysbiosis may be associated with acute gastrointestinal toxicity (Jang et al., 2023). The larger-scale prospective observational study “MicroLearner,” which enrolled 215 PCa patients undergoing radiotherapy, found that baseline fecal microbiota composition prior to radiotherapy could predict the risk of acute gastrointestinal toxicity; a decision tree model comprising genera such as *Faecalibacterium*, *Bacteroides*, *Parabacteroides*, *Alistipes*, *Prevotella*, and *Phascolarctobacterium* can be used to identify patients at high risk for post-radiotherapy gastrointestinal toxicity, suggesting that gut microbiota assessment may assist in the design of personalized radiotherapy regimens in the future (Ciorba et al., 2015; Iacovacci et al., 2024).

Regarding immunotherapy, although the overall response rate to PD-1/PD-L1 inhibitors in PCa is low, small-scale studies have observed an association between gut microbiota and response to immunotherapy. In metastatic castration-resistant prostate cancer (mCRPC) patients who received pembrolizumab following progression on enzalutamide, researchers analyzed pre-treatment gut microbiota composition and found no significant differences in overall microbiota composition between responders and non-responders. Furthermore, unlike in other cancer types, *A. muciniphila* did not exhibit higher abundance in responders, suggesting that microbiome characteristics associated with immunotherapy in PCa may be specific to the cancer type and treatment context (Peiffer et al., 2022). However, in other solid tumors, classic studies have shown that antibiotics can diminish the benefits of PD-1/PD-L1 therapy, while *A. muciniphila* abundance is associated with clinical benefit from immunotherapy. Oral administration of *A. muciniphila* can partially restore anti-PD-1 efficacy in mouse models, providing indirect insights into microbiota modulation in PCa immunotherapy (Routy et al., 2018).

Overall, the gut microbiota plays a role in various PCa treatment contexts. However, treatment-related microbiome findings should be interpreted in light of substantial clinical heterogeneity, including differences in treatment modality, treatment duration and prior systemic therapy. This bidirectional interaction, whereby treatment alters the microbiota and the microbiota in turn influences treatment response and toxicity, offers new research directions for precision therapy and supportive care in PCa. From a critical perspective, the evidence supporting microbiome-treatment interactions is uneven across treatment modalities. The association between gut microbiota and radiotherapy-induced gastrointestinal toxicity is supported by longitudinal and prospective observational data, making it one of the more clinically actionable areas. In contrast, evidence for microbiome modulation of immunotherapy response in PCa remains limited, partly because PCa generally shows a lower response rate to immune checkpoint inhibitors than several other solid tumors. Moreover, microbial signatures identified in other cancers, such as *A. muciniphila* enrichment in responders to PD-1/PD-L1 blockade, may not be directly transferable to PCa. For endocrine therapy, ADT and abiraterone clearly reshape the gut microbiome, but whether these microbial changes are causal mediators of treatment resistance or adaptive consequences of altered host androgen metabolism remains to be fully clarified. Therefore, future studies should integrate longitudinal microbiome profiling with treatment response, toxicity, circulating androgen levels, immune parameters, and metabolomic readouts.

## 5 Gut microbiota-targeted intervention strategies for prostate cancer

### 5.1 Diet, weight loss, and fatty acid interventions

Diet is the most practical intervention for modulating the gut microbiome. Raber et al. conducted an analysis of the association between diet and the microbiome in 79 PCa patients. The results indicated that dietary quality scores can predict certain characteristics of gut microbiota diversity and composition, confirming that diet remains a key modifiable factor for microbiota regulation in PCa patients (Raber et al., 2026). Frugé et al. found that in overweight or obese PCa patients, gut microbiota composition could change following preoperative weight loss or dietary intervention, and this change may be associated with tumor characteristics such as the Gleason score, suggesting that diet-microbiota interactions may participate in regulating the biological behavior of PCa (Fruge et al., 2018). Lachance et al. found that supplementation with omega-3 MAG-EPA could reduce PCa growth in mice and tumor progression in preoperative patients (Lachance et al., 2024). In addition to fatty acids, low-carbohydrate diets have also been incorporated into gut-related interventions for PCa. Based on the CAPS2 randomized clinical trial, Lin et al. found that low-carbohydrate diet/weight loss interventions can reduce body weight and improve the intestinal permeability marker zonulin. The results suggest that dietary interventions may influence the metabolic environment associated with PCa progression by improving intestinal barrier function (Lin et al., 2022).

### 5.2 Antibiotics, microbiota eradication, and FMT interventions

FMT, as an intervention to restore the gut microbiome, has recently been recognized as having the potential to modulate the gut-prostate axis. Pernigoni et al. found that FMT derived from HSPC could inhibit tumor growth in mice (Pernigoni et al., 2021). Lin et al. also found that FMT from healthy mice could slow tumor growth and reduce TNF-α levels (Lin et al., 2024). Terrisse et al. further demonstrated that FMT from healthy donors or co-housing could restore the antitumor efficacy of ADT (Terrisse et al., 2022). Although FMT has shown antitumor or ADT-modulating effects in mouse models, its application in PCa remains experimental. Potential risks include the transmission of enteric pathogens, viruses, and antimicrobial-resistant organisms, procedure-related complications, and the unintended transfer of unfavorable microbial functions. Future PCa trials should standardize donor eligibility, stool collection, batch traceability, administration route, dose and frequency. Screening panels should be adapted to local epidemiology and emerging infectious threats. Informed consent, prospective adverse-event monitoring, long-term follow-up, and predefined stopping criteria should also be incorporated. Until safety and efficacy are demonstrated in human PCa studies, FMT should be evaluated only under appropriate ethical and regulatory oversight and should not be considered part of routine PCa management.

### 5.3 Strain-specific, probiotic, and synbiotic-like interventions

Interventions targeting specific bacterial strains represent a direction with significant translational potential, with *A. muciniphila* and *P. stercorea* receiving the most attention. Pernigoni et al. found that administration of *P. stercorea* inhibited tumor growth in a castrated mouse model. Terrisse et al. found that oral administration of *Akkermansia* to PCa mice enhanced the antitumor effects of ADT. Yu et al. further found that *A. muciniphila* is enriched in ADT-sensitive CRPC patients, and its metabolite inosine can inhibit the development of castration resistance. These findings suggest that supplementation with specific bacterial strains may modulate endocrine resistance, and the “*Akkermansia-inosine*” axis may represent a new direction for CRPC intervention (Pernigoni et al., 2021; Terrisse et al., 2022; Yu et al., 2024).

There is currently limited evidence for probiotic interventions in humans, and their clinical role should presently be considered adjunctive rather than therapeutic. A randomised, double-blind, placebo-controlled trial of patients undergoing active surveillance for low-risk PCa showed that dietary supplements rich in phytochemicals slowed PSA progression. The addition of *Lactobacillus* probiotics led to a further improvement in PSA progression and inflammatory markers, suggesting that ‘phytochemicals + probiotics’ could be a milder adjunctive intervention for active surveillance of PCa (Thomas et al., 2026).

### 5.4 Interventions related to natural products, polyphenols, and gut microbial metabolites

Natural products and dietary polyphenols can be converted by the gut microbiota into bioactive metabolites, and thus have gradually attracted attention in PCa intervention studies. In a preoperative dietary intervention study, Gonzalez-Sarrias et al. administered walnuts or pomegranate juice, and found that the gut microbiota metabolites urolithin A glucuronide and small amounts of urolithin B glucuronide could be detected in prostate tissue from patients with benign prostatic hyperplasia (BPH) or PCa. This indicates that urolithin-like compounds produced by the metabolism of dietary tannins by gut bacteria can reach prostate tissue, providing early human evidence for the “dietary polyphenol–gut microbiota metabolite–local prostate action” pathway (Gonzalez-Sarrias et al., 2010). *In vitro* studies further demonstrated that gut microbiota metabolites such as urolithin A, urolithin B, and urolithin C can inhibit LNCaP cell proliferation and induce apoptosis. Additionally, some urolithins can influence dihydrotestosterone (DHT)-induced PSA secretion and its interaction with bicalutamide, suggesting that urolithin metabolites may affect androgen-related PCa signaling (Stanislawska et al., 2018). Subsequent studies also found that urolithin A and M4 valerolactone act on LNCaP cells, affecting cell proliferation, apoptosis, AR localization, Akt phosphorylation, and PSA secretion (Stanislawska et al., 2019); urolithin A can also inhibit the viability of PCa cells with different p53 statuses and induce apoptosis, suggesting that it may exert antitumor effects through pathways such as p53-MDM2 (Mohammed Saleem et al., 2020). Furthermore, extracts from *Epilobium* species and urolithins formed by gut microbiota conversion of epigallocatechin gallate can inhibit LNCaP cell proliferation, PSA secretion, and arginase activity (Stolarczyk et al., 2013).

There are also numerous animal studies demonstrating that natural products directly regulate the gut microbiota. Astragaloside IV-PESV stabilizes the gut microbiota and metabolites in PCa mice, reducing tumor volume and weight; FMT from the treatment group also inhibits tumor growth and downregulates the expression of RAGE, NF-κB, TNF-α, and IL-6 (You et al., 2024). Icaritin-curcumol (ICA-CUR) can alter gut microbiota and SCFA levels and enhance anti-tumor immunity of CD8+ T cells; FMT derived from the treatment group can also reduce tumor volume and weight (Xu et al., 2024). Icaritin can restore *Akkermansiaceae* and vitamin K2 levels and prolong survival time in TRAMP mice (Hu et al., 2026).

It is worth noting that randomized human trials of natural products do not consistently yield positive results. A multicenter, randomized, double-blind, placebo-controlled trial conducted by Mandl et al. evaluated the effects of muscadine grape skin extract (MPX) on biochemically recurrent PCa; the results showed that MPX did not significantly improve PSA slope, PSA doubling time, PSA response, or progression-free survival, and the study was terminated due to lack of efficacy (Mandl et al., 2025). However, microbiome analysis revealed increased relative abundances of *Roseburia faecis* and *A. muciniphila* in the MPX group, suggesting that natural products can alter certain beneficial microbial communities; however, such changes may not necessarily translate into clear clinical benefits.

Taken together, these studies represent distinct levels of evidence. Detection of microbiota-derived metabolites in human prostate tissue demonstrates exposure and biological availability, whereas *in vitro* and animal studies mainly establish mechanistic plausibility; clinical efficacy, however, must be determined in randomized trials, and the available randomized MPX trial did not demonstrate oncological benefit despite changes in potentially beneficial bacterial taxa.

### 5.5 Intervention of metabolites derived from the microbiome and bacterial steroid metabolic pathways

Butyrate is one of the typical SCFAs; *in vitro* studies have shown that sodium butyrate can induce cell death in DU145 and LNCaP cells, inhibit cell proliferation and migration, and increase methylglyoxal-related cytotoxicity by inhibiting the JAK2/STAT3/Nrf2/Glo1 pathway, suggesting that butyrate may have therapeutic potential in specific PCa cell contexts (Hsia et al., 2024). However, other studies have found that FMT derived from CRPC or SCFA supplementation may promote PCa progression (Liu et al., 2023). Therefore, future interventions targeting SCFAs should not simply aim to “increase” or “decrease” levels, but rather identify specific metabolites, target cells, and disease stages.

Bacterial steroid metabolic pathways represent a relatively new direction in PCa interventions, particularly for CRPC. *C. scindens* can convert cortisol into the potent androgen precursor 11β-hydroxyandrostenedione; metabolites in its conditioned medium promote LNCaP cell proliferation and migration and activate AR and AR-regulated genes, suggesting that targeting *C. scindens* or its androgen-producing pathway may help inhibit CRPC progression (Bui et al., 2023). Further studies have found that the desF gene of *C. scindens* is involved in androgen production, and that desF levels in the feces are elevated in patients who do not respond to abiraterone/prednisone therapy; bacteria originating from the urinary tract or prostate may also participate in steroid metabolism and promote AR-dependent PCa cell proliferation (Wang et al., 2025). These findings suggest that targeting steroid drug metabolism pathways may be a potential strategy to improve the response to endocrine therapy.

Overall, research on gut microbiota-targeted interventions for PCa remains in the early stages, with limited evidence from randomized controlled trials in humans. Most studies have been derived from animal experiments, FMT, dietary fatty acid modulation, supplementation with specific bacterial strains, natural product interventions, and studies on microbiota-associated metabolites. From a clinical translation perspective, these strategies should currently be regarded as adjunctive approaches rather than replacements for standard oncological therapy. Their future application will require clear patient selection criteria. In general, these strategies do not directly kill tumor cells but rather indirectly influence PCa onset, progression, castration resistance, or treatment response by reshaping the “gut microbiota–metabolite–inflammation/immunity/androgen” axis. Critically, microbiota-targeted interventions for PCa remain at an early translational stage. Although dietary modulation, FMT, probiotics, specific bacterial strains, natural products, and microbial metabolites have shown promise in preclinical models, robust evidence from randomized human trials is still scarce. Importantly, changes in microbial composition should not be equated with clinical efficacy. For example, some natural product interventions can increase potentially beneficial bacteria such as *A. muciniphila* or *R. faecis*, but these microbial shifts may not necessarily improve PSA kinetics, progression-free survival, or other clinically meaningful endpoints. In addition, FMT and probiotic-based strategies may have highly individualized effects depending on baseline microbiota composition, diet, host immunity, cancer stage, and concurrent therapy. Therefore, future intervention studies should clearly define the formulation, strain composition, dose, route, treatment duration, donor-screening criteria for FMT, baseline microbiota status, colonization or engraftment efficiency, safety monitoring, and clinically meaningful endpoints before these approaches can be recommended for routine PCa management.

## 6 Research limitations and challenges

Although existing studies have revealed multifaceted evidence linking the gut microbiota to PCa, the overall quality of evidence remains uneven and should be interpreted according to study design: prospective longitudinal human cohorts provide relatively high-level evidence for temporal and clinical associations; Mendelian randomization analyses provide moderate-level evidence for genetic causal inference; cross-sectional microbiome studies provide low-to-moderate-level evidence because they mainly identify associations rather than causality; animal models provide low-level evidence for direct human inference despite their mechanistic value; and cell-culture studies provide very-low-level evidence because they cannot reproduce the complex host–microbiota–tumor microenvironment. On the one hand, most studies are cross-sectional cohort or retrospective analyses, making it difficult to determine whether microbiota differences are a cause or a consequence of carcinogenesis. Discrepancies among study results may stem from factors such as small sample sizes, differences in patient ethnicity and dietary habits, variations in detection platforms (16S sequencing vs. metagenomic sequencing), and inconsistent data analysis methods (Salter et al., 2014). PCa patients often undergo various treatments, including ADT, radiotherapy, chemotherapy, and novel hormonal agents, and may also receive antibiotics, proton pump inhibitors, metformin, statins, or dietary supplements; however, these treatment- and medication-related confounders have not been uniformly recorded, adjusted for, or stratified in many microbiome studies. Furthermore, most studies focus on describing changes in microbiome structure, whereas functional validation using metagenomics, metatranscriptomics, metabolomics, targeted metabolite quantification, strain-level culture, or intervention-based experiments remains insufficient. Although animal models, FMT experiments, antibiotic interventions, and *in vitro* cell-culture studies are valuable for mechanistic exploration, their evidence level for direct clinical inference is relatively low because they cannot fully reproduce human microbial ecology, endocrine regulation, immune responses, and the prostate tumor microenvironment. Gut microbiome research also faces technical challenges, such as contamination issues and taxonomic annotation errors (Ward Grados et al., 2024). Although some studies have suggested the potential of the microbiome as a diagnostic or prognostic biomarker, these conclusions require validation in larger-scale prospective studies. Regarding clinical translation, interventions targeting the microbiome (such as FMT and probiotics) are feasible but require rigorous evaluation of safety and optimal protocols. In summary, research on the relationship between the gut microbiome and PCa is still in its early stages, and no definitive consensus has yet been reached.

## 7 Future research directions and outlook

First, large-scale, multicenter, prospective cohort studies should be conducted using standardized protocols for patient enrollment, dietary and medication recording, sample collection, DNA extraction, sequencing, bioinformatic analysis, and clinical stratification, so as to systematically collect gut microbiota, metabolomic, and immunomic data and investigate the causal relationship between microbiota changes and the onset/progression of PCa. By utilizing metagenomic, metatranscriptomic, and metabolomic technologies in conjunction with single-cell immunomics, we can elucidate the mechanisms underlying the interactions between microbial metabolites (such as SCFAs and microbial androgens) and host cellular signaling pathways. Additionally, humanized gut microbiota mouse models or organoid systems can be established to validate the direct effects of specific bacterial strains on tumors in a controlled environment. Regarding precision intervention strategies, it is necessary to screen for probiotic strains beneficial to PCa or design prebiotic formulations, and evaluate their impact on patient prognosis through randomized clinical trials. Multifactorial integration is also a growing trend, involving the combined analysis of microbiome characteristics with clinical pathology and blood biomarkers to establish comprehensive predictive models that optimize screening and treatment decisions. Some studies have already used machine learning to predict radiation therapy toxicity from microbiome data; similar methods can be extended to prognosis prediction and drug response. Finally, attention must be paid to individual variability and precision medicine, such as investigating the association between the microbiome and PCa in patients with different genetic backgrounds and lifestyles, with the aim of developing personalized microbiome intervention strategies. Overall, the most immediate translational priorities in PCa are to determine whether gut microbial features can predict progression from hormone-sensitive disease to CRPC, response to ADT or androgen-receptor pathway inhibitors, and radiotherapy-related gastrointestinal toxicity, and to evaluate microbiota-targeted interventions in clinically defined, stage- and treatment-stratified patient populations.

## 8 Conclusion

As a key component of the host’s external environment, the gut microbiota contributes to the development and progression of PCa through its metabolic products and immunomodulatory functions. In both basic and clinical research, the gut microbiota has been reported to be associated with androgen metabolism, pro-inflammatory signaling, and the tumor microenvironment, thereby potentially influencing PCa cell proliferation and treatment sensitivity. While multiple studies have elucidated the compositional characteristics of the gut microbiota in PCa patients and potential carcinogenic mechanisms, most findings still require validation in higher-level evidence settings, particularly prospective longitudinal human cohorts and reproducible multi-omics studies, because many current conclusions are derived from cross-sectional microbiome analyses and animal models experiments. In the future, leveraging emerging omics technologies and artificial intelligence, we can delve deeper into the multilevel interactions of the gut-prostate axis. Through a deeper understanding and regulation of the microbiota, we can open up new possibilities for personalized treatment and prevention of PCa.
